# Guided genetic screen to identify genes essential in the regeneration of hair cells and other tissues

**DOI:** 10.1038/s41536-018-0050-7

**Published:** 2018-06-04

**Authors:** Wuhong Pei, Lisha Xu, Sunny C. Huang, Kade Pettie, Jennifer Idol, Alberto Rissone, Erin Jimenez, Jason W. Sinclair, Claire Slevin, Gaurav K. Varshney, MaryPat Jones, Blake Carrington, Kevin Bishop, Haigen Huang, Raman Sood, Shuo Lin, Shawn M. Burgess

**Affiliations:** 1 Translational and Functional Genomics Branch, National Human Genome Research Institute, Bethesda, MD 20892 USA; 2 Functional and Chemical Genomics Research Program, Oklahoma Medical Research Foundation, Oklahoma City, OK 73104 USA; 3 Department of Molecular, Cell, and Developmental Biology, University of California Los Angeles, Los Angeles, CA 90095 USA

## Abstract

Regenerative medicine holds great promise for both degenerative diseases and traumatic tissue injury which represent significant challenges to the health care system. Hearing loss, which affects hundreds of millions of people worldwide, is caused primarily by a permanent loss of the mechanosensory receptors of the inner ear known as hair cells. This failure to regenerate hair cells after loss is limited to mammals, while all other non-mammalian vertebrates tested were able to completely regenerate these mechanosensory receptors after injury. To understand the mechanism of hair cell regeneration and its association with regeneration of other tissues, we performed a guided mutagenesis screen using zebrafish lateral line hair cells as a screening platform to identify genes that are essential for hair cell regeneration, and further investigated how genes essential for hair cell regeneration were involved in the regeneration of other tissues. We created genetic mutations either by retroviral insertion or CRISPR/Cas9 approaches, and developed a high-throughput screening pipeline for analyzing hair cell development and regeneration. We screened 254 gene mutations and identified 7 genes specifically affecting hair cell regeneration. These hair cell regeneration genes fell into distinct and somewhat surprising functional categories. By examining the regeneration of caudal fin and liver, we found these hair cell regeneration genes often also affected other types of tissue regeneration. Therefore, our results demonstrate guided screening is an effective approach to discover regeneration candidates, and hair cell regeneration is associated with other tissue regeneration.

## Identifying regenerative genes in non-mammalian vertebrates

A study on zebrafish has genetically screened 254 genes and identified 7 genes implicated in the development and regeneration of hair cells and other tissues. Humans and other mammals cannot regrow hair cells—inner-ear sensory receptors that enable hearing—whereas non-mammalian vertebrates, including zebrafish, can regrow these following injury. Researchers from the United States, led by the National Institutes of Health’s Shawn Burgess, screened adult zebrafish for genes active during the regeneration of inner-ear epithelium. The researchers then produced zebrafish without these genes to study their functions. The studies tested 254 genes known to respond during regeneration, and identified seven specifically impacting regeneration. Most of these seven genes also functioned in liver and fin tissue regeneration. Understanding the mechanisms of these genes may enable future research into regenerative therapies in humans.

## Introduction

Regenerative medicine offers promising potential to restore tissues and even whole-organs damaged by injury or disease conditions that afflict the daily life for millions of people worldwide.^1,2^ Developing effective regenerative medicine fundamentally relies on an advanced understanding of the biological processes that drive tissue repair and replacement. Several distinct processes are required for tissue regeneration, including initial damage signals, activation of local stem cells, response of the immune system, and initiation of regenerative proliferation.^3,4^ Genetic factors play a critical role in each of these processes. Although numerous genes and pathways are involved in tissue regeneration,^5,6^ there are great gaps in our understanding of which genetic factors are essential for the regeneration of a specific tissue.

Regenerative medicine holds a promise to improve the quality of life for millions of people suffering from hearing loss.^7,8^ Hearing loss is the most common congenital sensory impairment in newborns and the most common sensory deficit in the elderly. Most acquired hearing loss is from hair cell death exacerbated by genetic susceptibility, chronic exposure to ototoxic chemicals, and/or loud noise.^9^ Linkage studies and sequencing analysis indicate that genetic components contribute greatly to the onset of hearing loss (http://hereditaryhearingloss.org). However, it is challenging to identify genes and pathways that modulate susceptibility to hair cell loss in humans due to the difficulties in accessing the inner ear complex. More importantly, hearing loss in humans is permanent once it occurs, since humans have very limited potential to regenerate hair cells.^10^

In contrast to humans, non-mammalian vertebrates, including zebrafish, can regenerate lost hair cells and are constantly adding hair cells throughout their lives.^11–16^ Zebrafish are particularly suitable for studying hair cell death and regeneration because of their possession of skin-surface hair cells located in mechanosensory organs called neuromasts.^17–22^ Neuromast hair cells, like inner ear hair cells, can be damaged by ototoxic chemicals. Because of the skin-surface location, the regeneration of neuromast hair cells can be monitored using a fluorescent microscope and hair cell-specific vital dyes.^23,24^ Therefore, zebrafish serve as a valuable model system to study hair cell development and regeneration.

In addition to hair cell regeneration, zebrafish can regenerate many other tissues that humans and other mammals have very limited ability to regenerate, such as brain, heart, and limb/fin.^25,26^ The usefulness of zebrafish for tissue regeneration studies are further strengthened by the availability of its fully annotated genome sequence.^27^ The emergence of the genome-editing tool CRISPR/Cas9 has greatly accelerated the generation of zebrafish mutants and facilitates functional genomics studies.^28^ All these advantages, coupled with its simple husbandry and large number of offspring, make zebrafish a unique model for large-scale mutagenesis screening to systemically identify genetic factors that are essential for tissue regeneration.

In this study, we performed a guided mutagenesis screen utilizing zebrafish neuromast hair cell regeneration as a platform to identify genes essential for regeneration. We screened 254 stable gene mutations, setting up a screening pipeline for analyzing the general morphology, hair cell development, and hair cell regeneration of the mutants. We identified gene candidates that reveal surprising regeneration pathways. Further phenotyping of these candidate genes revealed both general pathways for regeneration as well as hair cell-specific pathways. This study broadens our understanding of the mechanism underlying tissue regeneration, providing important insights for regenerative medicine.

## Results

### Genes selected for screening

We performed a genetic screen of 254 gene mutations in zebrafish to systemically identify genes that are essential for hair cell development and regeneration. Most genes used for the initial screen were selected from the transcriptional profiling of the regeneration of adult zebrafish inner ear epithelium.^29^ The initial screen also included genes previously shown to be expressed specifically in inner ear and/or lateral line neuromasts, as well as genes relevant to hair cell development and human deafness. We expanded the gene selection categories as the screen progressed. When a positive candidate was identified, genes functionally relevant to the positive candidate were added to the screen. Supplemental Table 1 is a complete list of genes and mutations included in the screen.

### Mutation generation

Mutations in the target genes were generated by two mutagenesis approaches. Genes used for the early stages of the screen were mostly mutagenized by a retroviral insertional approach, carrying viral insertions in either exons or the 1st introns of candidate genes. A previous study has shown that insertion of retroviral DNA in exons and 1st introns have a high probability of being mutagenic.^30^ Consistently, our analysis showed many of these integrations resulted in a measurable reduction of mRNA expression (e.g., Suppl. Fig. 1a). However, some of the mutations caused no clear alteration in the transcription level (data not shown).

The emergence of CRISPR technology has greatly facilitated the generation of gene-specific mutations. Consequently, we shifted out strategy and generated mutations in more than 100 genes by CRISPR/Cas9 targeting and used these mutant fish for screening. The majority of the CRISPR/Cas9 mutations were frame-shift mutations in exons close to 5′ end of the gene. To improve the efficiency of CRISPR/Cas9-mediated mutagenesis, several strategies were implemented during screening. Accuracy of genome targeting was improved by using the NHGRI-1 zebrafish strain, a healthy and fecund strain derived from TAB-5 parents whose genomic DNA was extensively sequenced and variants documented.^31^ Highly active CRISPR/Cas9 targets were selected by coupling the NHGRI-1 variant data with our CRISPR prediction track or the CRISPRscan prediction software.^31,32^ Guide RNAs were synthesized in high-throughput fashion by a cloning-free assembly method.^33^ Mutation activity was measured shortly after injection in the founder embryos using CRISPR-STAT analysis in a 96-well plate format.^34^ In addition, multiplex targeting was applied by co-injecting 2–6 guide RNAs to accommodate the screening of multiple mutations simultaneously in the same animals. The in vivo activities of the CRISPR/Cas9 targets used for the screening were documented in the CRISPRz database,^35^ allowing reuse of the validated targets by other researchers and to help improve computational design in the future.

### Pipelines for mutation screening

**Fig. 1:**
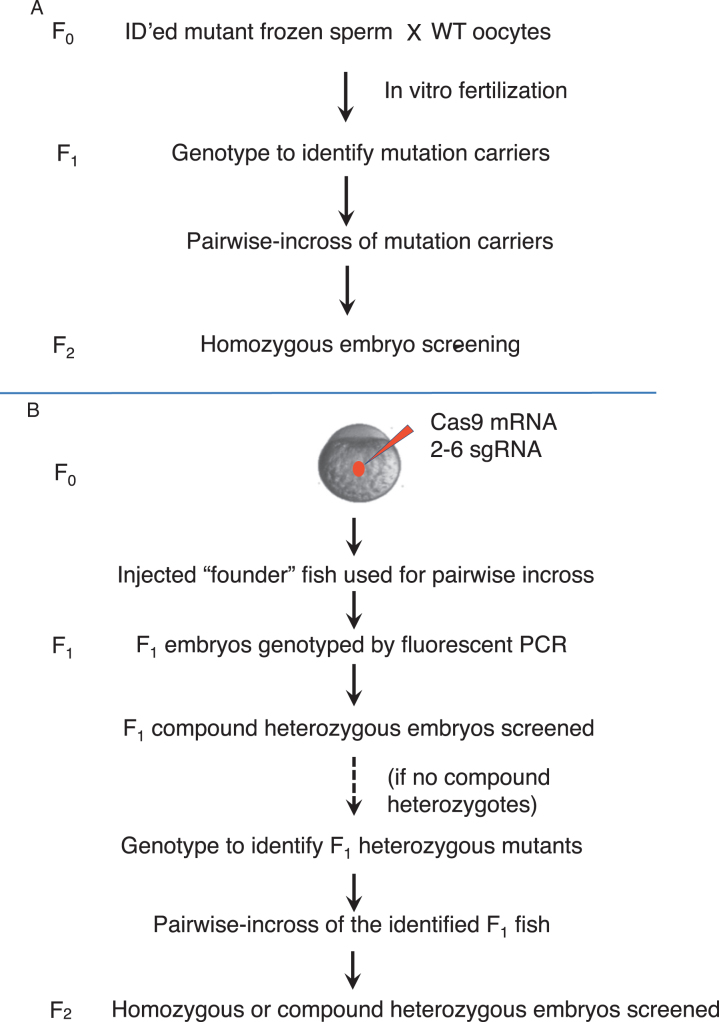
The screening pipelines. **a** The pipeline for screening mutations obtained by retroviral integration. Homozygous embryos from the F_2_ generation were used for screening. Mutations obtained by ENU or gene/protein trap were also screened using homozygotes from the F_2_ generation. **b** The pipeline for screening mutations obtained by CRISPR/Cas9. Multiplex targeting was usually used to generate CRISPR/Cas9 mutations, by co-injecting 2–6 single-guide RNA (sgRNA) together with Cas9 mRNA. Compound heterozygotes in the F_1_ generation were used for screening when the sgRNAs had sufficient measured somatic activity. Homozygotes or compound heterozygotes from the F_2_ generation were used when there were not enough compound heterozygotes found in the F_1_ generation

The screening was conducted as outlined in Fig. 1. For the retroviral insertional mutations, F_2_ embryos were primarily used for the screening (Fig. 1a). For CRISPR/Cas9-generated mutations, screening was conducted using either F_1_ embryos (when the CRISPR targets possess a relatively high activity), or F_2_ embryos (when the CRISPR targets possess a low activity) (Fig. 1b). Phenotypic analysis began with examination of the morphological phenotypes, followed by examination of the phenotypes in hair cell development and hair cell regeneration. The embryos used for phenotypic analysis were afterward genotyped to correlate phenotypes to homozygous or compound heterozygous mutations.

All of the mutations that caused a phenotypic alteration were re-screened to confirm the genotype–phenotype linkage, typically by analyzing mutations produced from a different pair of parents and by analyzing additional alleles.

### Genes affecting early embryonic development

**Fig. 2:**
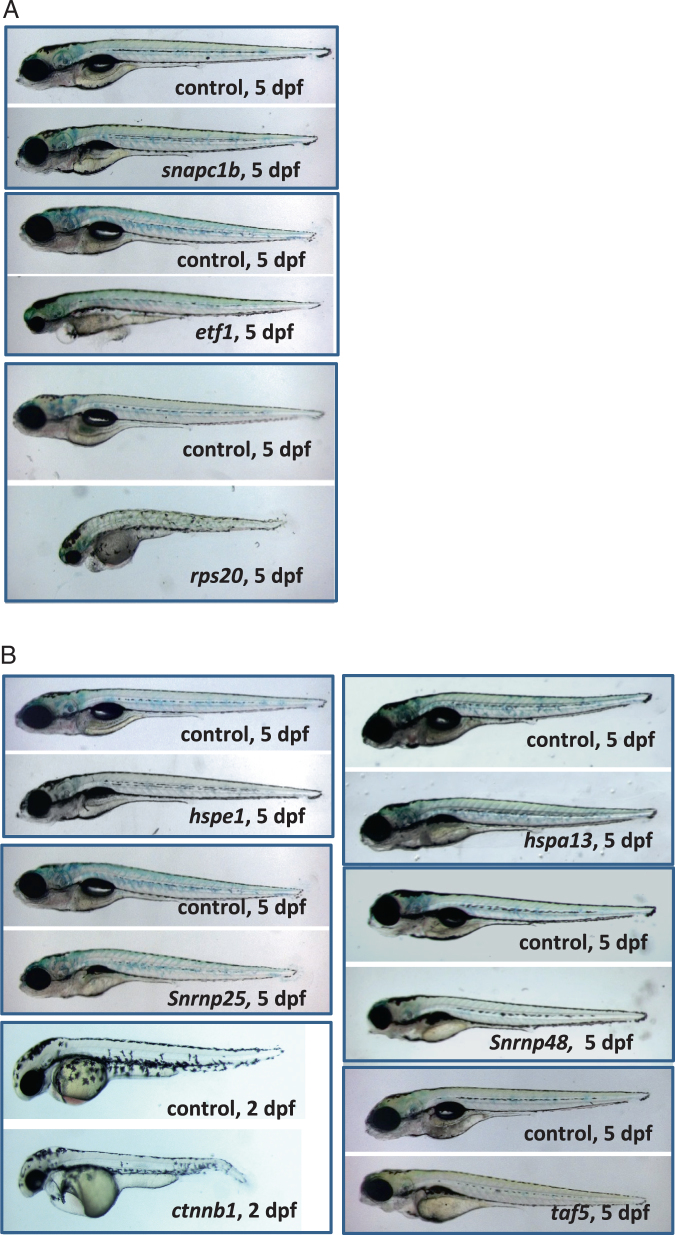
Mutations causing embryonic phenotypes. **a** Retroviral mutations causing embryonic phenotypes. Degree of severity ranged for the early embryonic phenotypes. *snapc1b* mutants appeared essentially normal but they failed to inflate the swim bladder and eventually die by day 10. *etf1* mutants had severe craniofacial defects, small eyes, and cardiac edema. *rps20* mutants were the most severely disrupted with severe growth retardation and an overall failure to thrive. **b** CRISPR/Cas9 mutations causing embryonic phenotypes. The mutants in heat shock family proteins *hspe1* and *hspa13* and small nuclear protein *snrnp25* all appeared normal but failed to inflate the swim bladder and eventually die around day 10–15. *snrnp48* mutants had small eyes and abnormal jaw structure. *taf5* mutants display craniofacial defects. β-catenin (*ctnnb1*) mutants have disrupted tail development, heart edemas and reduced head and eyes

Embryonic phenotypes were analyzed from 1 to 7 days post fertilization (dpf) for all the mutations screened. Mutations in 23 genes generated in this study affected early embryonic morphology (Suppl. Table 2). Among them, mutations in nine genes caused phenotypes that had not been reported for those genes previously (Fig. 2). We found that embryonic phenotypes that manifested between 1–2 dpf usually included small eyes, small head, and short body (e.g., mutations in *rps20* and *ctnnb1*). These mutants often died between 4–7 dpf. The phenotypes that manifested between 4–5 dpf usually included an un-inflated swim bladder, and these mutants usually died between 14–21 dpf (e.g., mutations in *snapc1b* and *hspe1*). Intermediate phenotypes and survival were observed for the mutations with phenotypes that manifested between 3–4 dpf (e.g., mutations in *etf1* and *taf5*).

### Genes affecting hair cell development

Hair cell development was analyzed using lateral line neuromasts at 5 dpf, a time right after the rapid growth of neuromast hair cells. Not surprisingly, we found mutations that manifested morphological phenotypes prior to 3 dpf caused a reduction in hair cell numbers, likely caused by the global developmental disruptions (Suppl. Fig. 2, Suppl. Table 2).

Mutations causing morphological phenotypes at 3 dpf or afterward had varying effects on hair cell development. Some of these mutations impaired hair cell development, while others showed no effect (Suppl. Figs. 2, 3, Suppl. Table 2). In addition, we found mutations in *prps1a* caused a reduction of hair cells at 3 dpf that recovered at 5 dpf (Suppl. Fig. 3). Consistent with the previously reported ENU-generated alleles,^36,37^ we found retroviral insertion in *lef1* gene caused a lack of tail neuromasts (data not shown).

High-magnification microscopic examination revealed some mutations caused an alteration in hair cell morphology. Mutation in *hdac1* caused a reduced condensation of hair cell nuclear DNA, in addition to a reduction in the number of hair cells (Suppl. Figs. 3, 4). Furthermore, we found a *mcoln1a* and *mcoln1b* double mutant possessed hair cells with abnormal nuclear morphology and various cytoplasmic abnormalities including fragmented mitochondria, enlarged lysosomes, and massive clustering of autophagosomes, with the detailed information published in our previous study.^38^

None of the mutations screened in this study caused an acceleration in hair cell development.

### Genes affecting hair cell regeneration

**Fig. 3:**
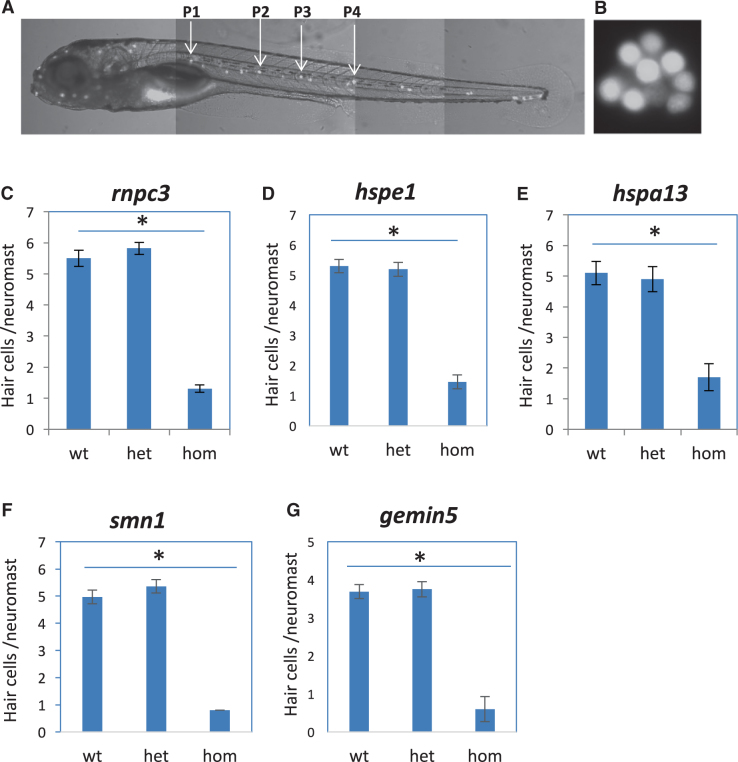
Mutations specifically affecting hair cell regeneration. **a** A fluorescent image of a zebrafish embryo at 5 dpf stained with Yopro-1, showing the distribution of neuromast hair cells. Arrows point to the P1–P4 neuromasts that were used for hair cell counting. **b** A magnified image of neuromast hair cells in the P1 neuromast. **c**–**g** Quantification of impaired hair cell regeneration caused by *rnpc3*^*la028632*^ retroviral insertional mutation (**c**), *hspe1*^*hg77*^ (6 bp CRISPR deletion) mutation (**d**), *hspa13*^*hg78*^ (12 bp CRISPR deletion) mutation (**e**), *smn1*^*fh299*^ (ENU missense Y262X) mutation (**f**), and *gemin5*^*hg80*^ (1 bp CRISPR deletion) mutation (**g**). The graphs are obtained from analyzing and then genotyping ~45 embryos generated from a pairwise incross of heterozygotes. Graphs show the mean and s.e.m. The difference between the wild-types and homozygotes is significant for all mutations (*p* < 0.05)

Hair cell regeneration was examined after morphological phenotypes and hair cell development had been determined. Hair cells were ablated by copper sulfate at 5 dpf and the regeneration of new hair cells was measured at 7 dpf. We found many of the mutations that impaired hair cell development also caused impaired hair cell regeneration, with the data from analyzing three such gene mutations shown in Suppl. Fig. 3. We identified seven genes that specifically affected hair cell regeneration (Fig. 3c–g and our published data^39,40^) without affecting hair cell development (Suppl. Fig. 5A–E). Surprisingly, these seven genes fell into three functional categories: three encoding heat shock proteins (*hspd1*, *hspe1*, *hspa1*3), three encoding RNA splicing factors (*rnpc3*, *smn1*, *gemin5*), and one encoding an N-glycosylation enzyme (*mgat5*). Whole-mount in situ hybridization analysis revealed only two of these seven genes, *rnpc3* and *mgat5*, were expressed in lateral line neuromasts (Suppl. Fig. 5F–I and our published data^39,40^), indicating neuromast-specific expression in the uninjured state is not necessary for causing a hair cell regeneration-specific phenotype.

Mutations in four of these genes (*hspd1*, *hspe1*, *hspa13*, and *rnpc3*) showed no major morphological defects other than an un-inflated swim bladder at 5 dpf (Fig. 2b).^40,41^ Homozygous mutant embryos for *smn1* and *gemin5* were indistinguishable from wild-type embryos at 5 dpf, but failed to survive to adulthood (data not shown).^42^ Mutation in *mgat5* caused no embryonic phenotypes and adult homozygotes were generally healthy and fertile.^39^ Mutations in *mgat5* mildly promoted hair cell regeneration,^39^ while mutations in the other six genes strongly inhibited hair cell regeneration (Fig. 3c–g and our previous findings).^40^

To confirm the morphological and hair cell phenotypes were specifically due to the genetic mutations being studied, we created a 2nd allele for *hspe1*, *hspa13*, and *gemin5* with CRISPR/Cas9. We found these additional alleles reproduced the morphological and regeneration phenotypes (Suppl. Fig. 6). We confirmed the regeneration phenotype by ablating hair cells with neomycin (data not shown).

### Effect of hair cell regeneration genes on the regeneration of fin and liver

**Fig. 4:**
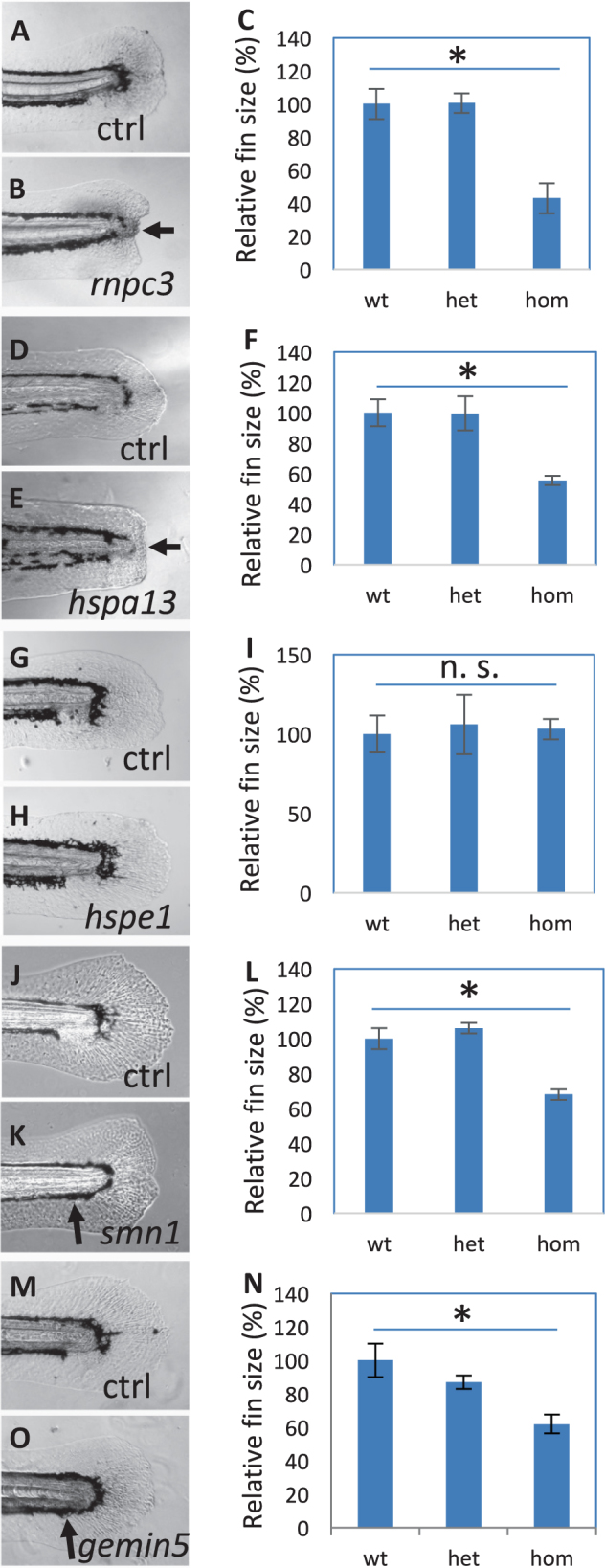
Fin regeneration in the mutants affecting hair cell regeneration. **a**–**c** Regenerated fins in the control (**a**) and *rnpc3*^*la028632*^ (**b**), and the quantification of the fin areas (**c**). **d**–**f** Regenerated fins in the control (**d**) and *hspa13*^*hg78*^ mutants (**e**), and the quantification of the fin areas (**f**). **g**–**i** Regenerated fins in the control (**g**) and *hspe1*^*hg77*^ mutant (**h**), and the quantification of the fin areas (**i**). **j**–**l** Regenerated fins in the control (**j**) and *smn1*^*fh299*^ mutant (**k**), and the quantification of the fin areas (**l**). **m**–**o** Regenerated fins in the control (**m**) and *gemin5*^*hg80*^ mutant (**n**), and the quantification of the fin areas (**o**). Arrows in **b** and **e** point to a lack of caudal fin fold in *rnpc3* and *hspa13* mutants. Arrows in **k** and **o** point to a lack of ventral pigment gap in *smn1* and *gemin5* mutants. Graphs are generated by analyzing more than eight embryos per genotype. Error bars show the s.e.m. There was no difference in the regeneration between wild-type and heterozygotes for all the mutations, so control (Ctrl) is used to represent both. There is a significant difference between the wild-type and mutant embryos for mutations in *rnpc3*, *hspa13*, *smn1*, and *gemin5* (*p* < 0.05 for all), but not for mutation in *hspe1* (n. s.)

In addition to hair cell regeneration, zebrafish can regenerate many other tissues that humans and other mammals cannot regenerate. Our previous studies have shown that inhibiting the function of *hspd1* and *mgat5* genes not only affect hair cell regeneration but also fin regeneration.^39,40^ To understand whether the other genes involved in hair cell regeneration also impact other tissues’ ability to regenerate, we first examined the development and regeneration of the median fin fold in the mutants. Although none of these mutations affected fin development (data not shown), they affected fin regeneration to a varying extent (Fig. 4): *rnpc3* and *hspa13* mutations caused a severe inhibition as evident by a lack of median fin fold growth, a phenotype that was also observed in the *hspd1* mutant;^40^ *hspe1* mutations showed no apparent effect on fin regeneration; *smn1* and *gemin5* mutations caused an inhibition that was often visualized as an elimination of ventral pigment gap.

**Fig. 5:**
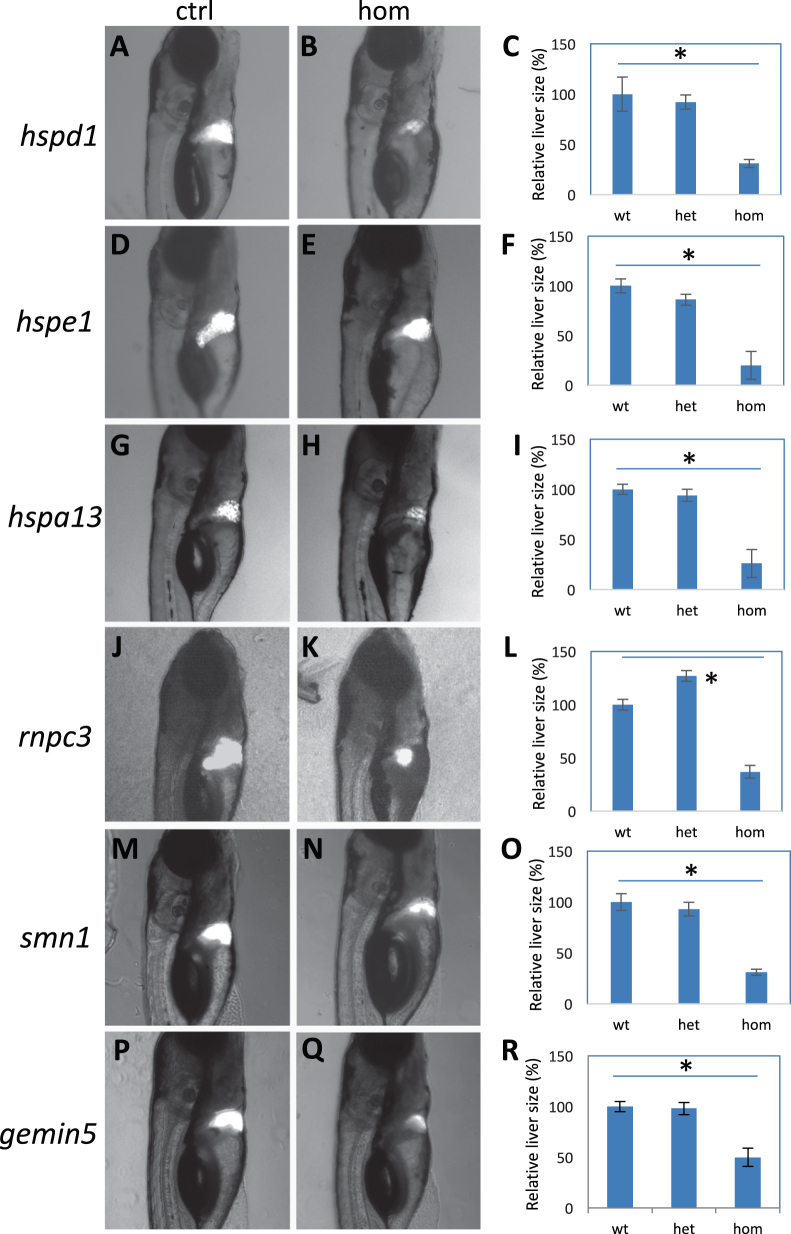
Liver regeneration in the mutants. **a**–**c** Regenerated liver in the control (**a**) and *hspd1*^*la026911*^ mutant (**b**), and the quantification of the liver areas (**c**). **d**–**f** Regenerated liver in the control (**d**) and *hspe1*^*hg77*^ mutant (**e**), and the quantification of the liver areas (**f**). **g**–**i** Regenerated liver in the control (**g**) and *hspa13*^*hg78*^ mutant (**h**), and the quantification of the liver areas (**i**). **j**–**l** Regenerated liver in the control (**j**) and *rnpc3*^*la028632*^ mutant (**k**), and the quantification of the liver areas (**l**). **m**–**o** Regenerated liver in the control (**m**) and *smn1*^*fh299*^ mutant (**n**), and the quantification of the liver areas (**o**). **p**–**r** Regenerated liver in the control (**p**) and *gemin5*^*hg80*^ mutant (**q**), and the quantification of the liver areas (**r**). Graphs are generated from analysis of approximately 45 CFP-positive embryos obtained by crossing one fish carrying heterozygous gene mutation with the other fish carrying heterozygous mutation and an allele of Tg(*fabp10*:CFP-NTR). Error bars show the s.e.m. Control (Ctrl) represents both wild-type and heterozygotes, as there was no difference observed between them. A significant difference was observed between the wild-type and the mutant for all the mutations (*p* < 0.05 for all)

We further examined the effect of inhibiting these hair cell regeneration genes on the development and regeneration of the liver, using a liver-specific labeling and ablation transgene system Tg(*fabp10*:CFP-NTR) as a readout.^43^ We found liver development was modestly impaired in *hspd1*, *hspe1*, and *rnpc3* mutants, and appeared to be normal in *hspa13*, *smn1*, and *gemin5* mutants (Suppl. Fig. 7). However, all six mutations caused a strong inhibition of liver regeneration (Fig. 5).

Altogether these data demonstrate that these genes play a stronger role in regeneration than in development, and furthermore indicate that there are both shared and specific pathways underlying the regeneration of hair cells, fins, and livers.

### Injury-induced expression is associated with regeneration

**Fig. 6:**
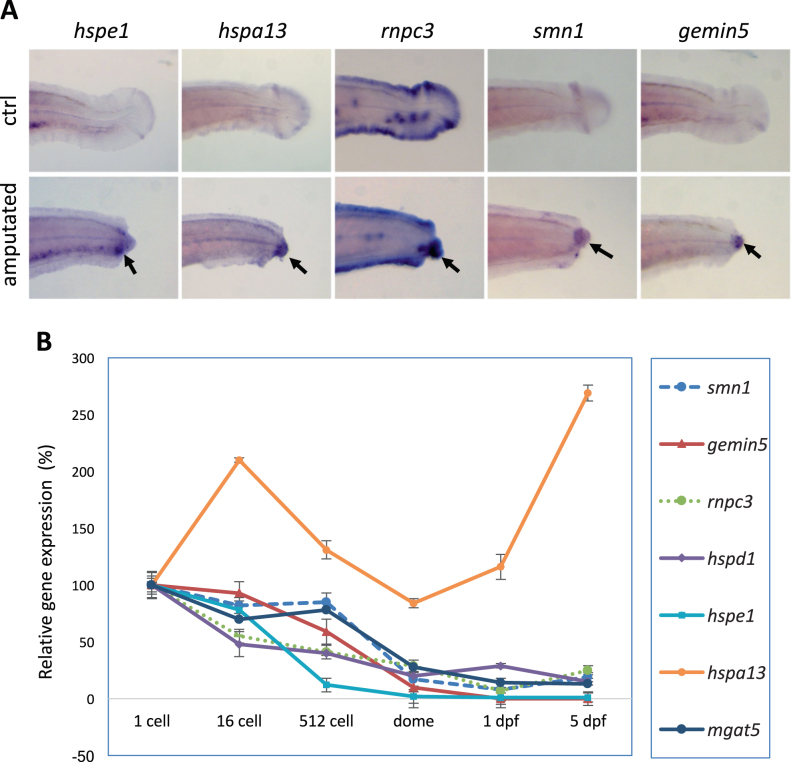
Injury-induced expression is associated with regeneration. **a** Whole-mount in situ analysis of gene expression after fin amputation. Arrows point to the injury-induced expression at the amputation sites. TAB-5 wild-type embryos at 3 dpf were used for fin amputation, and the amputated embryos were fixed at 15 h post amputation for whole-mount in situ analysis. TAB-5 wild-type embryos at 4 dpf without amputation were used as the control. **b** Quantitative PCR analysis of gene expression during maternal stages (1-cell, 16-cell), maternal–zygotic transition (512-cell), and later stages (dome, 1 dpf, and 5 dpf). Beta-actin was used as internal reference. The sequences for the primers used for the probe synthesis and qPCR are listed in Suppl. Table 3

We tested how an injury affects the expression of these identified regeneration genes. We induced the injury response by fin amputation and evaluated expression of each gene by whole-mount in situ hybridization analysis. We observed an obvious induction of expression of *hspe1*, *hspa13*, *rnpc3*, *smn1*, and *gemin5* genes at the amputation site (Fig. 6a), consistent with our previous observation of injury-induced expression of *hspd1.*^40^ Upregulation of these genes at the injury site indicates a responsiveness of these genes during regeneration and as their absence in the mutants implies their function is necessary for initiation of the regeneration response.

Maternally deposited RNAs and proteins could cause a delay in the manifestation of mutant phenotypes potentially masking roles these genes have in early developmental processes. We were unable to evaluate the contribution of maternally deposited proteins to early development as antibodies were not commercially available. As an alternative, we measured the levels of transcripts of the seven regeneration genes at six different embryonic stages, including two maternal stages (1-cell and 16-cell), one stage during maternal and zygotic transition (MZT, 512-cell), one stage at the end of the MZT (dome), and two zygotic stages (1 dpf and 5 dpf). Quantitative RT-PCR analysis revealed these seven genes possessed different expression dynamics (Fig. 6b), but most genes were strongly downregulated at the mRNA level days before the regeneration tests were performed. We cannot completely rule out developmental phenotypes masked by residual wild-type protein, but the whole-mount in situ hybridation and quantitative PCR (qPCR) results strongly suggest there is a function for each of these genes that is directly related to wound healing or regeneration.

## Discussion

Using zebrafish genetics to study regeneration allows us to identify genes involved in wound healing processes that are no longer available to mammals. A large-scale genetic screen provides a platform for the unbiased identification of novel genes or novel functions of known genes that are essential for tissue regeneration.

In this study, by taking advantages of the skin-surface location of zebrafish neuromast hair cells and the advancement in genome editing technologies, we conducted a genetic screen on hair cell regeneration involving 254 stable genetic mutations. We identified seven genes that are essential for hair cell regeneration but not required for normal hair cell development. Surprisingly, these seven regeneration genes fall into three functional categories: heat shock proteins (*hspd1*, *hspe1*, and *hspa13*), RNA splicing factors (*rnpc3*, *gem5*, and *smn1*), and a glycosylation enzyme (*mgat5*). Further analysis of these seven genes revealed that some but not all have similar effects on the regeneration of caudal fin and liver. These data indicate the regeneration of a specific tissue requires both tissue-specific and general regeneration-associated genes.

The recovery rate of this screen was 2.8% (7/254). We believe this is a significant enrichment for identifying genes of interest. If the original data set of approximately 2100 genes were to be screened to completion, we would expect to identify >60 regeneration-specific genes. It is important to note that neither the magnitude of transcriptional change from the original data set, nor pathway models of which genes would be most likely to impact regeneration were significantly good predictors for identifying key regeneration genes. This confirms our initial belief that genetic screening remains an important tool in identifying the important pathways of any biological process.

Large-scale screening of random zebrafish mutants show that roughly 6–7% of all zebrafish genes have an obvious morphological defect when mutated.^44^ Our data is consistent with this observation (23 of 254) suggesting most of our mutations resulted in significant loss-of-function. However, it is likely that the recovery rate would have been slightly higher if all screened genes were confirmed true loss-of-function mutations. We examined the knockdown efficiency for many of the screened mutations using semi-quantitative RT-PCR analysis. We found that some mutations, either from retroviral insertion or CRISPR mutagenesis, reduced the quantity of mRNA of the targeted gene presumably through nonsense-mediated decay, strongly indicating these mutations are disrupting normal function. However, the existence of undocumented and/or mutation-induced alternative splicing could compensate for any given mutation. RT-PCR analysis also showed there were retroviral or CRISPR mutations that caused no significant alteration in the mRNA levels. Some of these mutations (typically the CRISPR/Cas9-generated ones) still showed homozygous phenotypes (e.g., the *hspe1* mutation in Suppl. Fig. 1B), but many did not. These findings indicate that transcriptional analysis is not sufficient to evaluate knockdown or knockout efficiency. A more accurate evaluation for these mutations will depend on an advancement in the availability of antibodies against zebrafish proteins. Nevertheless, it would not be surprising that some of these mutations, especially the ones causing no alteration in gene expression, did not, in fact, interfere with gene function. It is also very likely that some mutations cause a partial loss-of-function that was not sufficient to alter hair cell regeneration.

The seven positive hits identified in this study, including one enhancer and six inhibitors of regeneration, belong to three functional categories. Our previous study has shown that *hspd1* contributes to tissue regeneration by acting as an extracellular injury signal to stimulate an immune response,^40^ while *mgat5* modulates cell signaling through post-translational glycosylation of intracellular and extracellular proteins.^39^ Hair cell regeneration was also be inhibited by the mutation *phoenix*, a gene of unknown function.^45^ Consistently, chemical compounds that are capable of modulating hair cell regeneration possess diversified structures and mediate biological processes ranging from signaling transduction, immune response, and histone modification.^46–48^ Altogether these findings indicate hair cell regeneration is a multifaceted process, regulated by a variety of biological activities.

An in-depth investigation is required to understand the mechanistic role of each of the candidates during tissue regeneration, and to elucidate how tissue-specific environment and modulators influence the regeneration outcome. Future efforts should also be directed to study their regenerative potential in other model systems. Conserved regenerative function has been observed for various genetic factors across different vertebrate models.^40,49,50^ It would not be surprising if understanding the regeneration mechanism of these seven positive hits could provide valuable guidance for the strategic design and development of regenerative medicine for restoring injured or diseased tissues in humans.

## Methods

### Zebrafish husbandry and embryology

Zebrafish embryos were obtained from natural crosses and staged according to Kimmel et al.,^51^ in compliance with NIH guidelines for animal handling and research (Protocol G-01-3). All protocols and methods related to animals or animal tissues were approved by animal care and use committee of the National Human Genome Research Institute (protocol #G-01-3). Whole-mount in situ hybridization was performed on TAB-5 wild-type embryos that were treated with 0.003% of phenylthiourea to inhibit pigmentation. qPCR analysis was performed using SYBR™ Green PCR Master Mix (Thermo Fisher Scientific, Cat #4344463), with cDNA template synthesized from total RNA of TAB-5 wild-type embryos collected at six developmental stages: 1-cell, 16-cell, 512-cell, dome, 1 dpf, and 5 dpf. Primers used for in situ probe synthesis and qPCR analysis were designed to target different exons of the same genes, to eliminate the interference of genomic DNA. The sequences of the primers are listed in Suppl. Table 3.

### Mutation generation

Retroviral insertional mutations were generated as previously reported.^52^ In brief, in vitro fertilization was performed by using oocytes from wild-type TAB5 zebrafish females and cryo-preserved sperm samples carrying the desired mutations. The resulted F_1_ fish were genotyped to verify the mutations and then used for producing an F_2_ generation which was used for phenotypic screening. All retroviral insertional mutations were genotyped using allele-specific primers for PCR and agarose gel analysis.

CRISPR mutations were generated as described.^33^ In brief, CRISPR targets were selected by combining NHGRI-1 and CRISPRscan target site predictions.^31,32^ Cas9 mRNA was synthesized using mMesage mMachine (Ambion) and guide RNA synthesized using HiScribe T7 High Yield RNA Synthesis Kit (NEB). Founder fish were generated by injecting 150 pg of Cas9 mRNA and 50 pg of guide RNA into NHGRI-1 embryos. Mutation was detected by fluorescent-based PCR fragment analysis.

### Knockdown efficiency analysis

Knockdown efficiency was analyzed by semi-quantitative RT-PCR with beta-actin as an internal reference. In brief, 16–20 embryos from a single heterozygous incross or an injected founder fish incross were collected individually in Trizol at 3 dpf, and then used for genomic DNA and total RNA extraction. The extracted genomic DNA was used for genotyping and the extracted total RNA was used for mRNA expression analysis by semi-qPCR. All gels were derived from the same experiment and were processed in parallel.

### Morphological phenotype analysis

Embryos from a single heterozygous incross or a CRISPR founder fish incross were used for morphological phenotype analysis, from 1 dpf to 7 dpf. The analyzed embryos were genotyped afterward to study genotype and phenotype correlation.

### Hair cell development analysis

Hair cell development was analyzed at 5 dpf, using 40–50 embryos obtained from a single pair heterozygote incross or a CRISPR founder fish incross. The embryos were stained with Yopro-1 (Molecular Probe, Cat #Y3603), and orientated to a lateral view in 96-well plate for counting hair cells in the lateral line neuromasts using a fluorescent microscope. Hair cells in the P1–P4 neuromasts were counted for each embryo.^53^ The analyzed embryos were afterward genotyped, to obtain the average number of hair cells per neuromast in the mutants and control siblings.

### Immunostaining

Immunostaining of neuromasts was conducted as previously described.^53^ In brief, embryos obtained from a cross of a single pair of heterozygotes were fixed with 4% PFA at 5 dpf. Hair cells were stained with a combination of two monoclonal antibodies specific to hair cells, myosin-VIIa (Developmental Studies Hybridoma Bank, MYO7A 138-1, 1 µg/ml) and hair cell soma-1 (Developmental Studies Hybridoma Bank, HCS-1, 1 µg/ml), and a secondary antibody conjugated with Alexa 488 (Invitrogen, A11001, 4 µg/ml). Nuclei of the neuromasts were stained by DAPI (Thermo Fisher Scientific, D1306). The stained embryos were embedded in low-melting agarose and imaged using a confocal microscope.

### Hair cell regeneration analysis

Approximately 45 embryos obtained from a heterozygote incross or a CRISPR founder fish incross were used for hair cell ablation and then regeneration analysis. Hair cells were ablated at 5 dpf by a 2-h treatment with 10 µM copper sulfate (Sigma, Cat #451657). The hair cell ablated embryos were recovered for 48 h and then used for hair cell regeneration analysis. The regenerated hair cells were stained by Yopro-1. The average number of the regenerated hair cells were obtained from counting P1–P4 lateral line neuromasts. The analyzed embryos were genotyped to determine linkage to the phenotype.

### Fin development and regeneration analysis

Embryos obtained from a heterozygote incross were anesthetized and used for the analysis. Fin development was determined at 5 dpf. For the regeneration analysis, the median fin fold was amputated at 3 dpf. The amputation was positioned at the posterior end of ventral pigmentation break, and the anterior end was used as a landmark for analyzing regeneration. The regeneration was imaged at 7 dpf. ImageJ was used for outlining and measuring the area of the regenerated fin. The analyzed embryos were then genotyped. The quantification data were obtained from analyzing 10 embryos per genotype.

### Liver development and regeneration analysis

The liver development and regeneration was analyzed using a liver-specific nitroreductase-mediated ablation system Tg(*fabp10*:CFP-NTR).^43^ The embryos used for the analysis were obtained by crossing a pair of fish, one carrying the heterozygous gene mutation, the other carrying the heterozygous mutation and an allele of Tg(*fabp10*:CFP-NTR). Approximately 45 CFP-positive embryos were used for each analysis. Liver development was analyzed at 5 dpf. For liver regeneration analyses, the embryos at 3 dpf were subjected to liver ablation using 10 mM of metronidazole for 1.5 days. Liver regeneration was then analyzed at 7 dpf, by imaging the ablated embryos from a lateral view with head facing right. ImageJ was used to measure the area of the livers. All the analyzed embryos were genotyped afterward. For data quantification, approximately 10 embryos were used for each genotype.

### Statistical analysis

The statistics were conducted using ANOVA (for comparison of multiple means) or two-tailed Student’s *t*-test (for comparision of two means). A difference was considered as significant when the *p-*value was less than 0.05. Bar graphs showed the mean and the standard error of the mean (s.e.m.). All experiments shown were replicated at least two times that produced consistent results.

### Data availability

All data generated or analyzed during this study are included in this published article (and its Supplementary Information files). CRISPR guide sequences also available at https://research.nhgri.nih.gov/CRISPRz/.

## Electronic supplementary material

Suppl fig Legends

Supplemental figure 1

Supplemental figure 2

Supplemental figure 3

Supplemental figure 4

Supplemental figure 5

Supplemental figure 6

Supplemental figure 7

Supplemental figure 8

Supplemental table 1

Supplemental table 2

Supplemental table 3
