# Supplementary material for: Guided genetic screen to identify genes essential in the regeneration of hair cells and other tissues
Source: NPJ Regen Med. 2018 Jun 4;3:11. doi: 10.1038/s41536-018-0050-7 (PMC5986822; doi:10.1038/s41536-018-0050-7)
Supplement: Supplementary file 1 — Suppl fig Legends [file 41536_2018_50_MOESM1_ESM.docx]

**Supplementary figure legends:**

Suppl. Fig. 1 Semi-quantitative PCR analysis of hair cell regeneration mutations.

Semi-quantitative RT-PCR was used to analyze the gene expression in the homozygous embryos carrying the *rnpc3^la028632^* retroviral insertional mutation (A), the *hspe1^hg76^* (10 bp deletion) mutation (B), and the *gemin5^hg81^* (20 bp deletion) (C). The primers used are as indicated. β-actin is used as an internal reference. The *rnpc3* and *gemin5* mRNA’s show clear evidence of reduction, but the *hspe1* mutation did not impact mRNA levels. All gels derive from the same experiment and were processed in parallel.

Suppl. Fig. 2 Mutations tested that affected hair cell development.

(A-D) Developmental timing of neuromast hair cell formation was assayed by Yopro-1 staining (A-C) or hair cell-specific antibody staining (D). Arrows point to hair cell-positive neuromasts. Mutant embryos had smaller staining area for each neuromast and fewer neuromasts, indicating an impairment in hair cell development. (E-H) Quantification of neuromast hair cell development by counting Yopro-1 positive hair cells. Compared to the control (Ctrl) siblings, homozygous (hom) mutants of sec61a1show a significant reduction in the average number of hair cells per neuromast.

Suppl. Fig. 3 Mutations tested that affected hair cell development as well as hair cell regeneration.

(A-C) Quantification of the development and regeneration of neuromast hair cells in the mutants homozygous for *snrnp25* (A), *hdac1* (B), and *prps1a* (C). Hair cell development was assayed at 5 dpf (except otherwise indicated). Hair cell regeneration was assayed by copper ablation at 5 dpf and regeneration at 7 dpf (labeled on graph as “regen”). Graph shows the mean and s.e.m. Control (ctrl) represents both wild type and heterozygotes, as no difference was observed between them. The difference between control and homozygous mutants is indicated. Asterisks indicate the difference is significant (p < 0.05). n. s. indicates the difference is not significant.

Suppl. Fig. 4 *hdac1* mutation causes abnormal hair cell morphology.

(A-F) Confocal imaging of immunofluorescently-stained neuromasts in the control (A-C) and *hdac1* mutant (D-F) embryos. Hair cells were stained in green with hair cell-specific antibodies to myo6 and myo7, neuromast nuclear DNA were stained in blue using DAPI. C and F are the magnified images of the areas boxed in B and E, respectively. White arrows in C and F point to the morphology of hair cell nuclear DNA, which is more condensed in the control embryo (C) than in the mutant embryo (F). Scale bar, 10 μm in A, B, D and E; 5 μm in C and F.

Suppl. Fig. 5 Hair cell development in the regeneration-specific mutants and the expression patterns of the identified genes.

(A-E) Quantification of hair cell development in the mutant embryos carrying the *rnpc3^la028632^* retroviral insertional mutation (A), *hspe1^hg76^* (6 bp deletion) mutation (B), *hspa13^hg78^* (12 bp deletion) mutation (C), *smn1^fh299^* (ENU Y262X) mutation (D), and *gemin5^hg80^* (1 bp deletion) mutation (E). Approximately 10 embryos were analyzed for each data point. The graphs show the mean and s.e.m. The difference between the wild-types and homozygous mutants was not significant (n. s.) for all the mutations, although there was a modest reduction trend in *hspa13*. (F-J) Whole-mount in situ analysis of the expression of the regeneration genes in TAB-5 wild-type embryos at 4 dpf. Arrows in F-J point to the expression in liver. Arrowheads in J point to the expression of *rnpc3* in lateral line neuromasts.

Suppl. Fig. 6 Impaired hair cell regeneration validated with a second mutant allele for *hspe1*, *hspa13* and *gemin5*.

Quantification of hair cell regeneration in the mutant embryos carrying *hspe1^hg76^* (10 bp deletion) mutation (A), *hspa13^hg79^* (6 bp deletion) mutation (B), and *gemin5^hg81^* (20 bp deletion) mutation (C). No difference was observed between the wild-types and heterozygous carriers, so their data were pooled as control (ctrl). There is a significant difference between the control and the homozygotes for all the mutations (p<0.05).

Suppl. Fig. 7 Liver development in the mutants affecting hair cell regeneration.

Quantification of liver development in the mutant embryos carrying the *hspd1^la026911^* mutation (A), *hspe1^hg77^* mutation (B), *hspa13^hg78^* mutation (C), *rnpc3^la028632^* (D), *smn1^fh299^* mutation (E), and *gemin5^hg80^* mutation (F). The graph is obtained from analyzing approximately 45 CFP-positive embryos from one parent carrying a heterozygous mutation crossed with the other parent carrying a heterozygous mutation and an allele of Tg(*fabp10*:CFP-NTR). Graphs show the mean and s.e.m. Asterisks indicate a significant difference. n. s. indicates a difference that is not significant.

Suppl. Fig. 8 Unprocessed DNA agarose gel images.

Unprocessed images showing the knockdown efficiency of mutations in *rnpc3^la028632^* (A), *hspe1^hg77^* (B)and *gemin5^hg80^* (C). MW stands for molecular weight. Bp stands for base pairs. ctrl stands for wild-type or heterozygotes, since there was no difference observed between them. Hom stands for homozygotes. Magnified areas in white dashed boxes are shown in Suppl. Figure. 1.  All gels derive from the same experiment and were processed in parallel.

**Supplementary tables:**

Suppl. Table 1 Genes and mutations screened.

A total of 254 gene mutation alleles were screened, including 213 genes screened by a single mutation allele, 15 genes screened by two individual mutation alleles, and 11 screened for double or triple mutations. Almost all mutations with an la# or CRISPRz ID# were generated for this screen. For retroviral insertional mutations, la# was assigned according to Varshney GK, et al ([42](#_ENREF_42)), and the inserted retroviral DNA is approximately 6 kb in length ([44](#_ENREF_44)). For CRISPR mutations, CRISPRz ID# was assigned as in Varshney GK, et al. ([25](#_ENREF_25)). For mutations made by CRISPR/Cas9, many different CRISPR mutation alleles were analyzed for each gene to verify the mutant phenotypes, however, only the representative mutation allele for each gene is shown in the table. Homozygous mutations were used for screening most of the genes (“del” indicates deletion mutations, and “ins” indicates insertion mutations). Compound heterozygotes (indicated as "compound") were used for screening for some of the genes. Additional reference information for most genes and mutations can be found in ZFIN website (<http://zfin.org/>). Additional information for mutations with a CRISPRz ID# can be found in the CRISPRz database (<https://research.nhgri.nih.gov/CRISPRz/>). Additional information for GBT protein trap mutations can be found at <http://zfishbook.org/> ([45](#_ENREF_45)). Detailed information for *mcoln1a*, *mcoln1b* and *mcoln1ab* mutations have been reported in our previous study ([38](#_ENREF_29)).

Suppl. Table 2 Mutations affecting embryonic development.

A total of 23 gene mutations was found in this screen causing embryonic phenotypes. The details of each gene mutation can be found in Suppl. Table 1. Among them, 11 gene mutations produced phenotypes similar to those observed in alleles reported in the ZFIN website (<http://zfin.org/>), 3 produced phenotypes that have been reported in our previous studies, and 9 produced novel phenotypes that are shown in Fig. 2. In addition to the major embryonic morphological phenotypes, the effects of these mutations on hair cell development and regeneration are also summarized in this table.

Suppl. Table 3 Sequences of the primers used for in situ probe synthesis and qPCR.

The forward and reverse primers for each gene are located in different exons to eliminate the amplification of genomic DNA. For the primers used for in situ probe synthesis, T3 and T7 promoter sequences were added to the 5-terminus of the forward and reverse primers, respectively, to generate sense and antisense probes. The length of the probes ranges from 200 to 600 bp. For the primers used for qPCR analysis, the resulting amplicons have a length between 150 to 200 bp.
