# Supplementary figures and images for: Guided genetic screen to identify genes essential in the regeneration of hair cells and other tissues
Source: NPJ Regen Med. 2018 Jun 4;3:11. doi: 10.1038/s41536-018-0050-7 (PMC5986822; doi:10.1038/s41536-018-0050-7)

## Suppl. Fig. 1

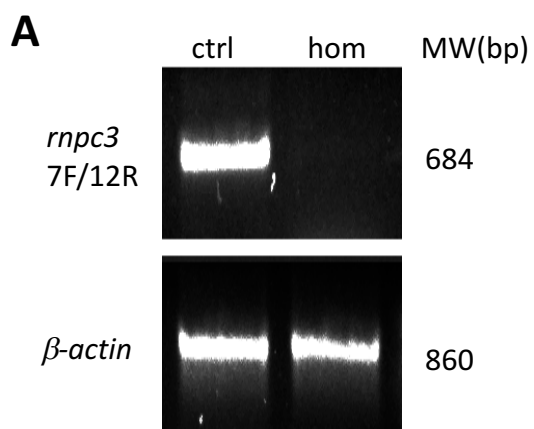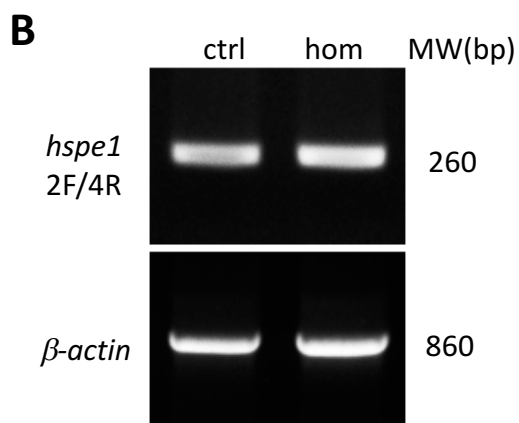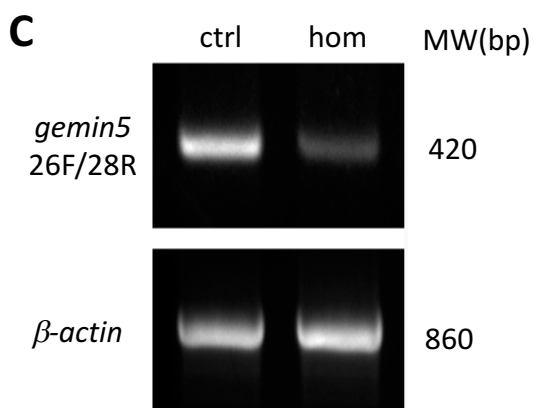

Supplement: Supplementary file 2 — Supplemental figure 1 [file 41536_2018_50_MOESM2_ESM.pdf]

## Suppl. Fig. 2

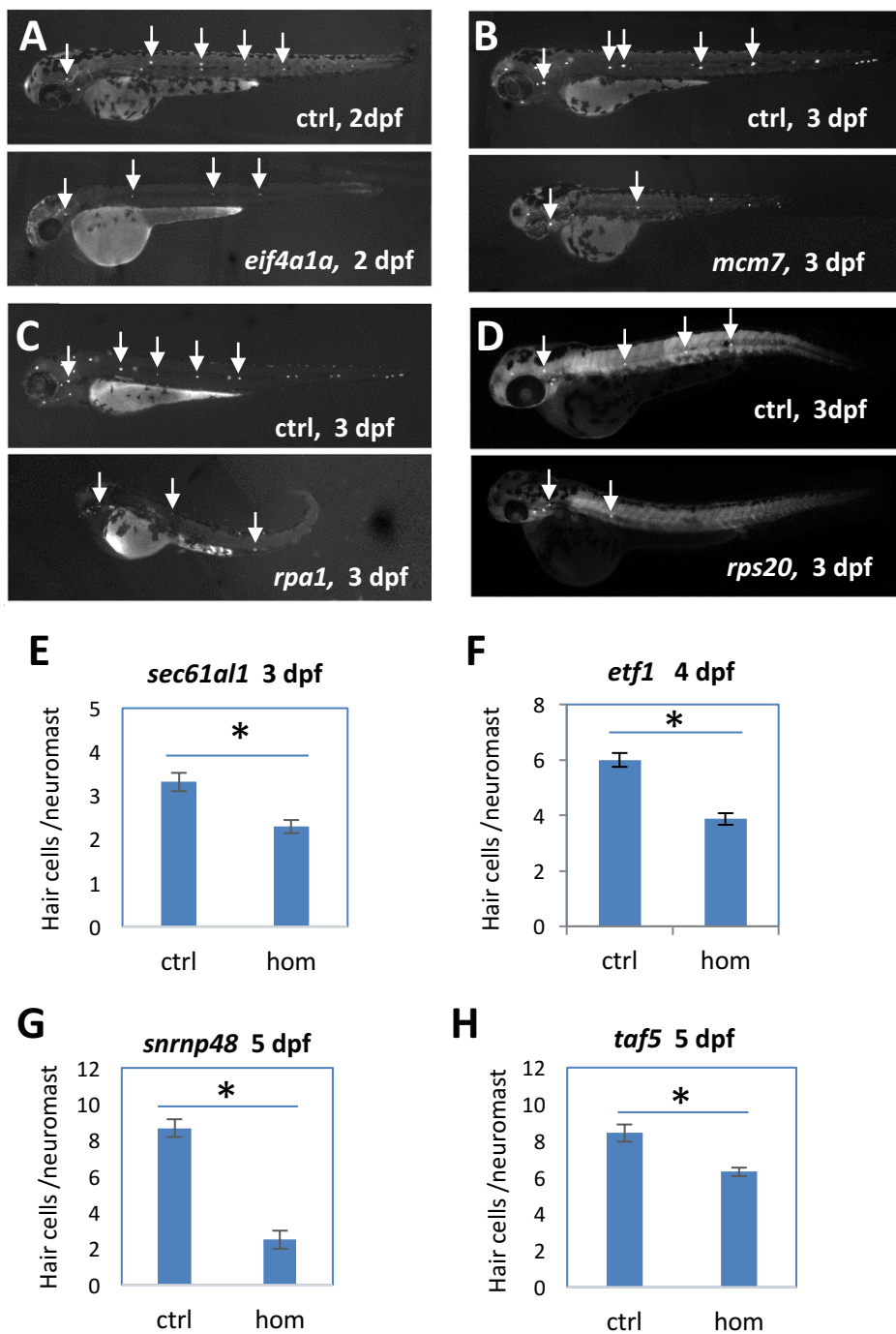

Supplement: Supplementary file 3 — Supplemental figure 2 [file 41536_2018_50_MOESM3_ESM.pdf]

Suppl. Fig. 3

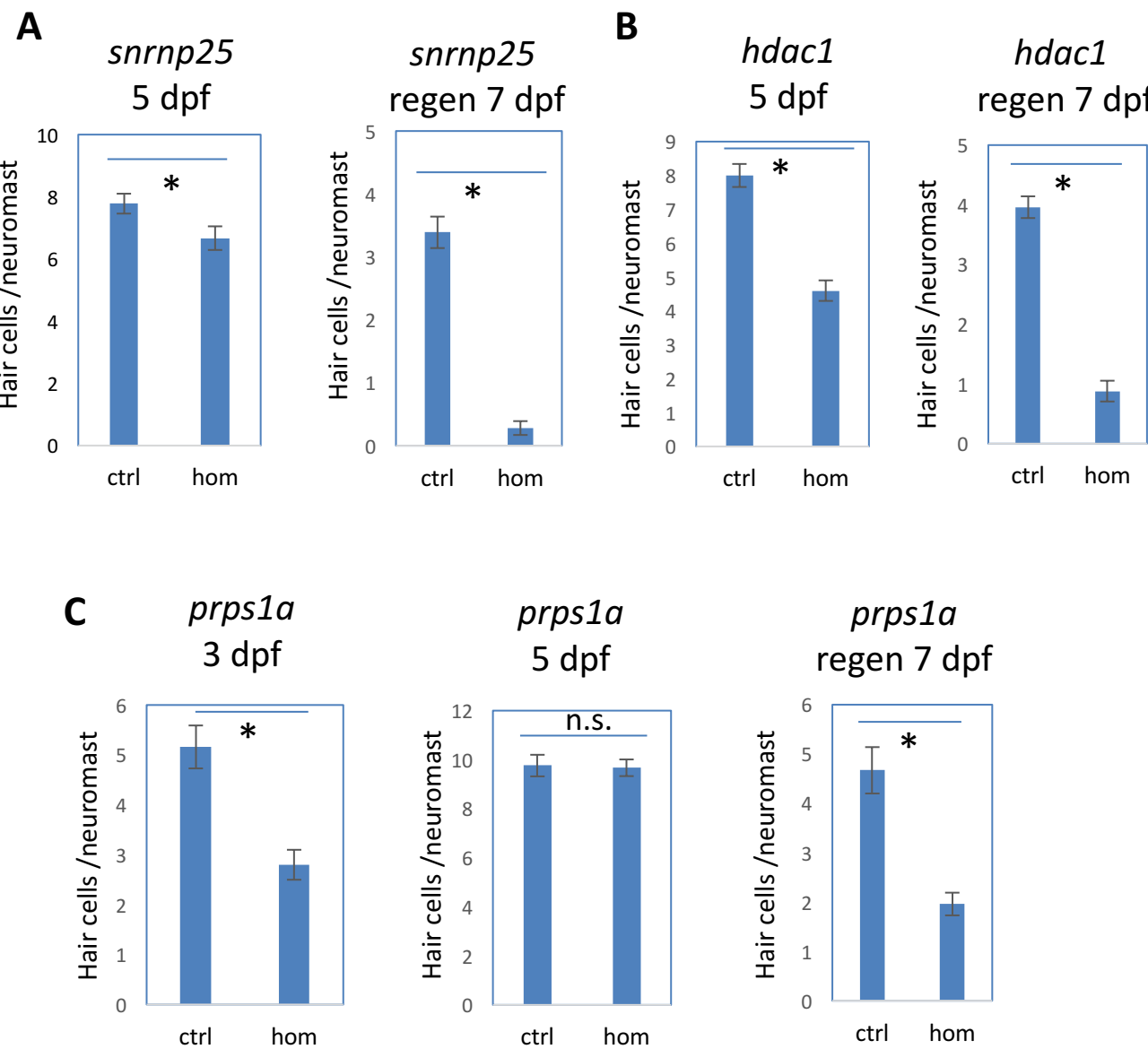

Supplement: Supplementary file 4 — Supplemental figure 3 [file 41536_2018_50_MOESM4_ESM.pdf]

Suppl. Fig. 4

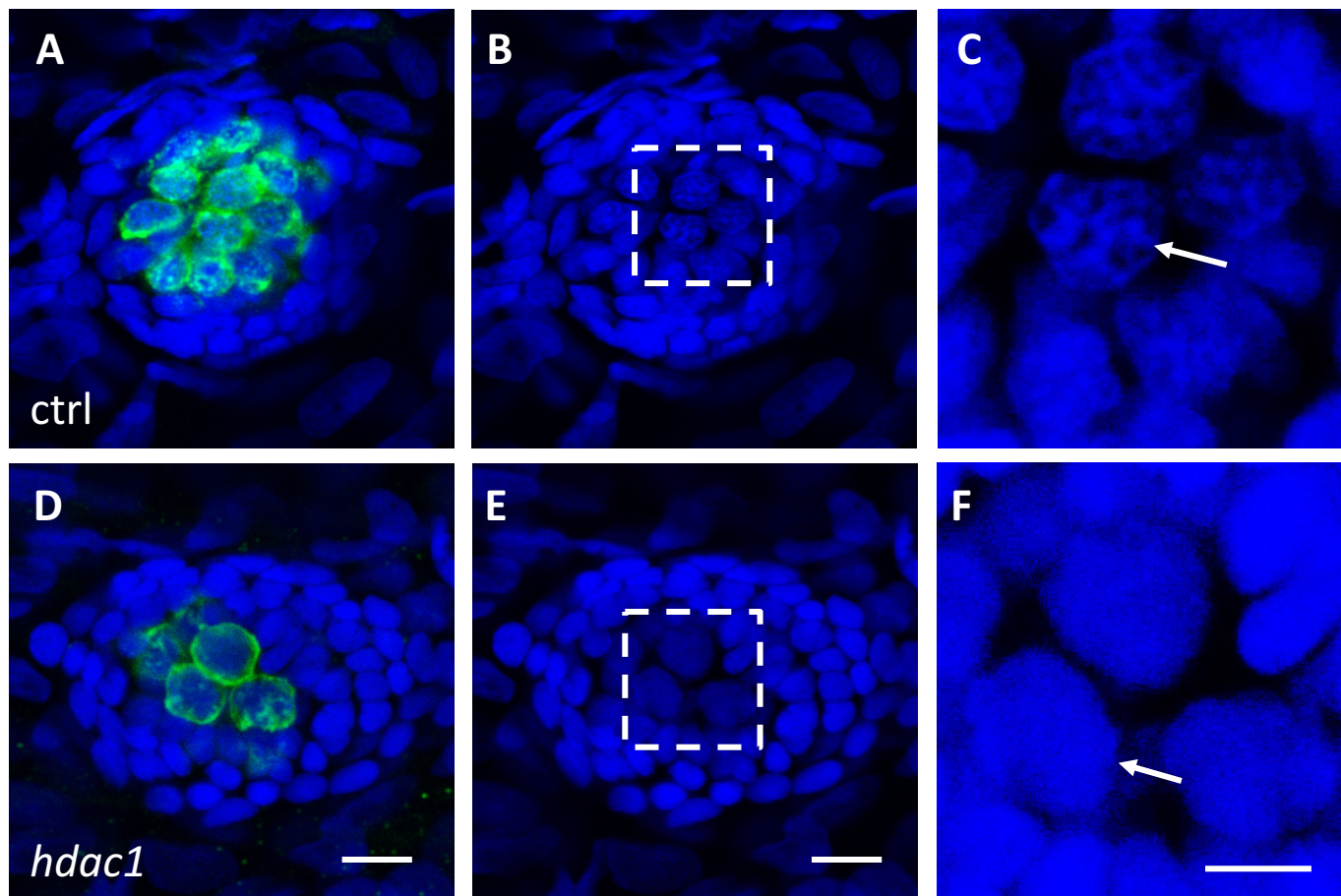

Supplement: Supplementary file 5 — Supplemental figure 4 [file 41536_2018_50_MOESM5_ESM.pdf]

Suppl. Fig. 5

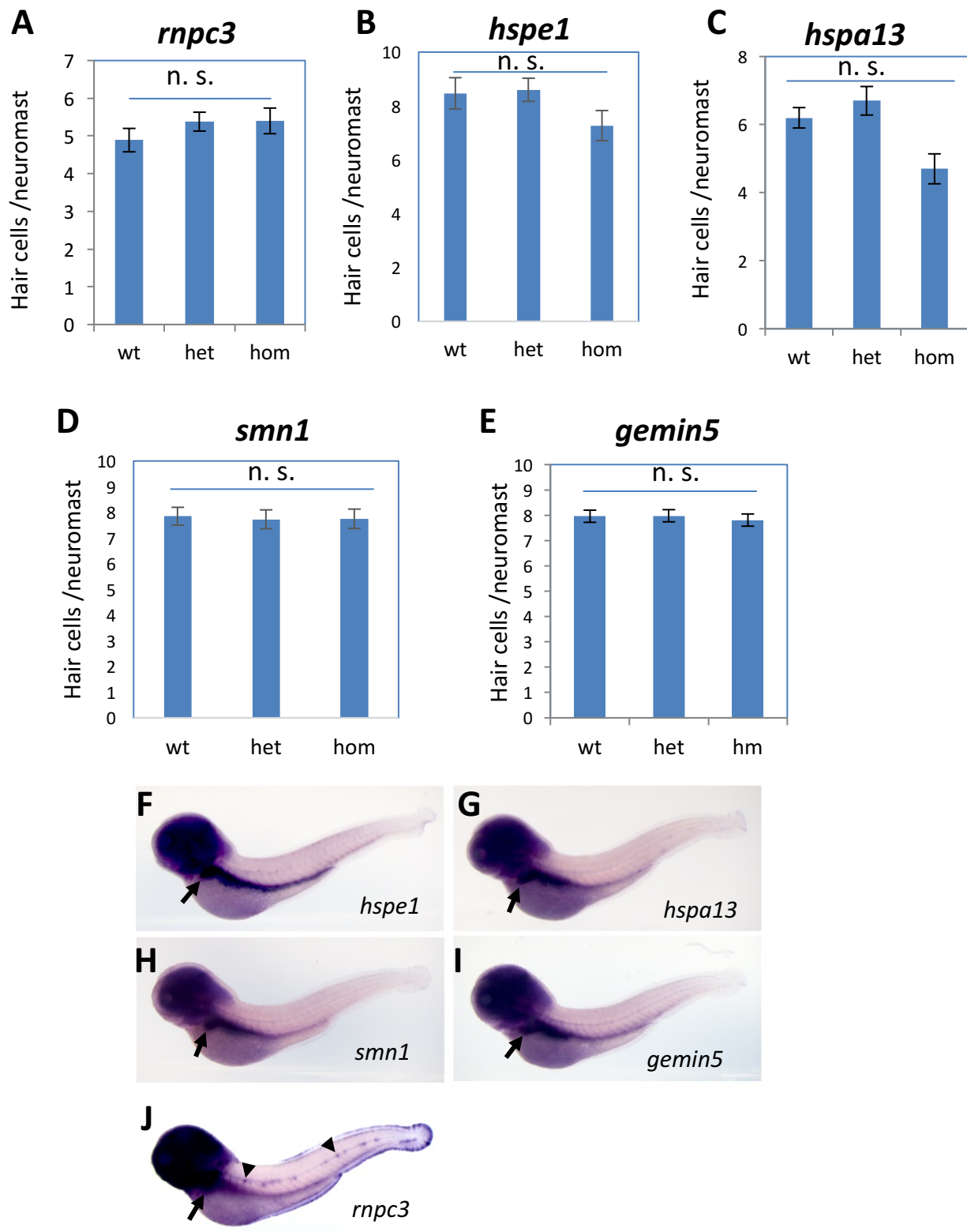

Supplement: Supplementary file 6 — Supplemental figure 5 [file 41536_2018_50_MOESM6_ESM.pdf]

Suppl. Fig. 6

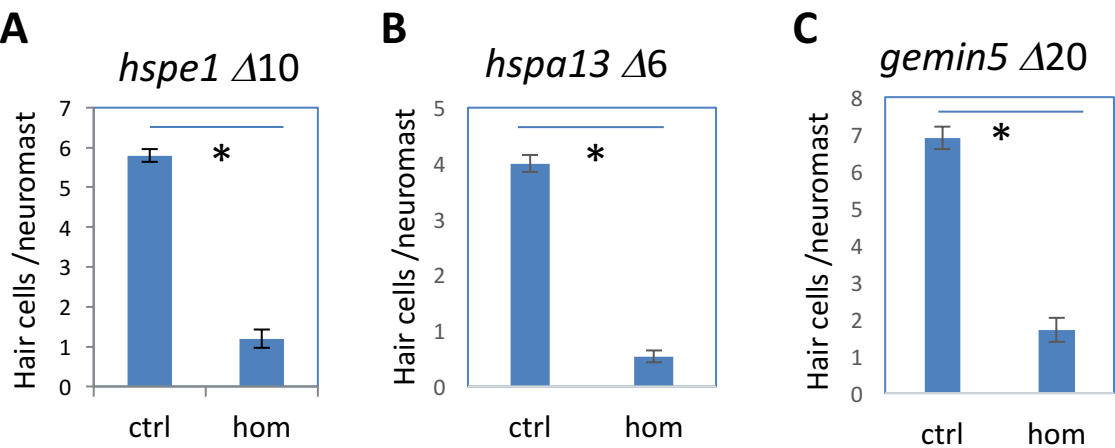

Supplement: Supplementary file 7 — Supplemental figure 6 [file 41536_2018_50_MOESM7_ESM.pdf]

Suppl. Fig. 7

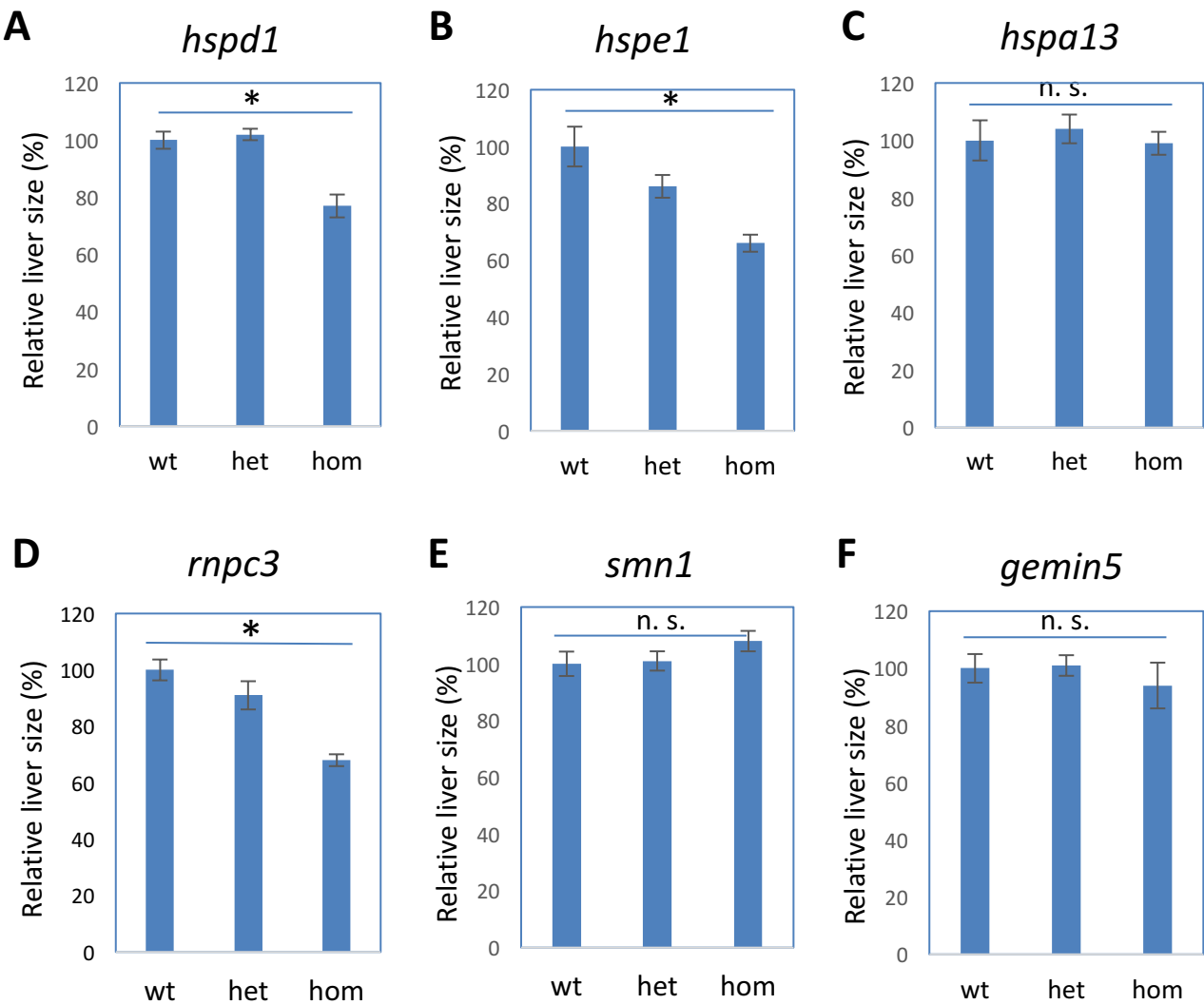

Supplement: Supplementary file 8 — Supplemental figure 7 [file 41536_2018_50_MOESM8_ESM.pdf]

Suppl. Fig. 8

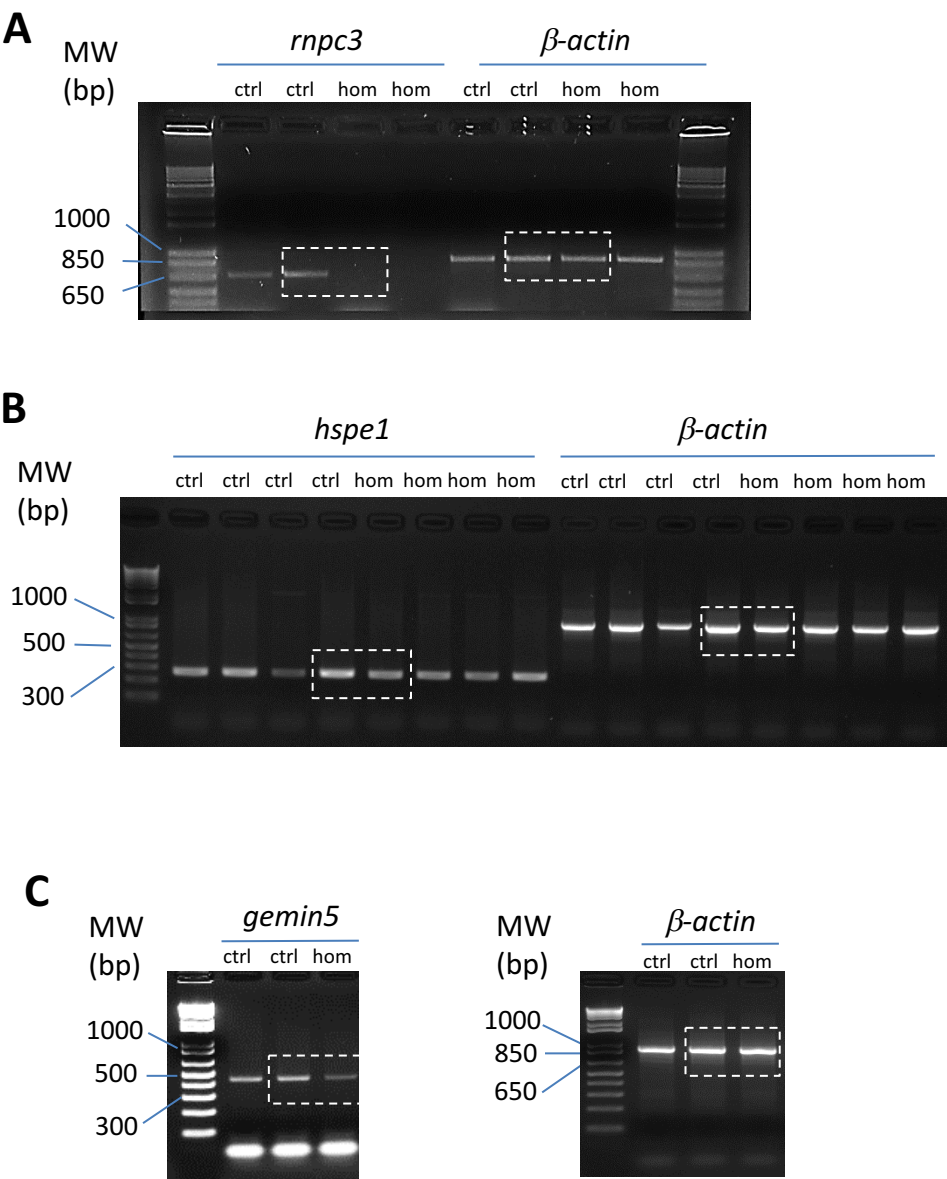

Supplement: Supplementary file 9 — Supplemental figure 8 [file 41536_2018_50_MOESM9_ESM.pdf]
